# 

**DOI:** 10.1192/bjb.2025.12

**Published:** 2026-02

**Authors:** Femi Oyebode

**Affiliations:** School of Psychology, University of Birmingham, UK. Email: femi_oyebode@msn.com

**Figure:**
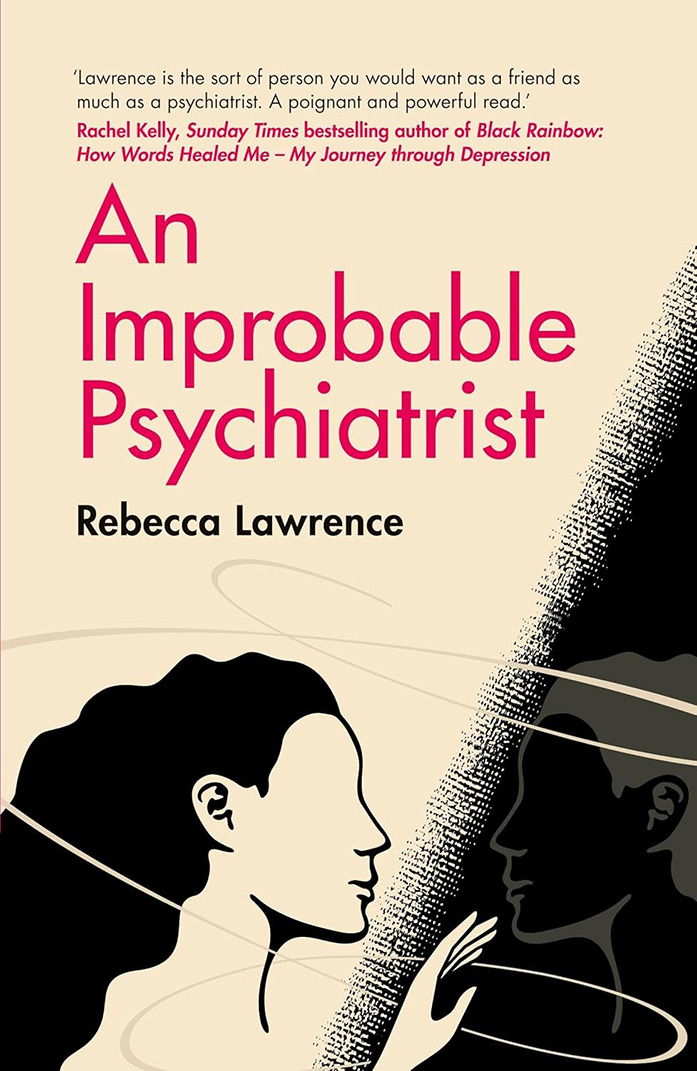

This is an account of a life, not a memoir of mental illness. It depicts with clarity, uncommon honesty and an uncompromising forthrightness Rebecca Lawrence's experience of mental illness within her life. Her voice is in a particular register that suits her story: it is often tentative, fragile, vulnerable, timid even. But there is steel there, too, a deep reserve of determination and inner strength that makes it possible for her to face adversity, to re-emerge after disturbing and distressing events and to carry on to new challenges. It may be trite to say that she triumphed, but it is true to remark that she succeeded despite her travails.

Lawrence entered medical school at the age of 17 years. We learn of what it was like to be a medical student, of the transition from student to junior doctor, of training first as a general practitioner and then as a psychiatrist and of becoming a consultant psychiatrist. She tells of her marriage and supportive husband, her abortion, stillbirth, several miscarriages and the birth of her three daughters. Then there is how mental illness, episodes of depression with psychosis, inflected her life, pervading and distorting it, often rendering her existence perilous and ruinous. It is an extraordinary and gripping account of how to live life despite grave mental illness.

Shame, a sense of being exposed to the scrutiny of others because of her experience of mental illness, and of wanting to hide or disappear, of feeling fundamentally flawed and unworthy, is at the heart of her account. She says of one of her admissions, ‘I see a group of visiting doctors, people who had been in my year at medical school. They are psychiatrists, and don't expect to see me. I see sudden surprise and pity, and most of all embarrassment. I'm not a person anymore, and I'm definitely not a doctor. I'm a patient, a psychiatric patient. I feel the bitterest shame at my continuing existence and want only not to be myself. Each bit of me, my voice, my skin, my space – they are all repulsive to me’ (p. 4). The role of shame in shaping personal, subjective responses to mental illness is not discussed as much as it should be within psychiatry, nor is its role in deepening despair and leading towards suicide.

Lawrence's account highlights the imprecision of diagnostic practice and treatments in psychiatry. She draws attention to how hard it is for the patient to accept clinical diagnoses and how persevering with treatment following recovery of sorts is influenced by side-effects. The impact of doctor–patient relationships on trust, hope and well-being is exemplified by her long-term relationship with her psychiatrist, Professor Blackwood. There are, too, the difficulties inherent in how the poorly resourced mental health services are unable to respond promptly and flexibly to crises.

This is a book that ought to be widely read. It shines light on how severe mental illness can potentially blight life but in this case how life can flourish despite the destructive incursions of mental illness. Finally, it demonstrates that one can continue to be cherished by loved ones despite the toxic and wearing effects of mental illness on relationships.

